# Sequential Philadelphia‐Positive Pre‐B Acute Lymphoblastic Leukemia and Chronic Myeloid Leukemia With Distinct BCR::ABL1 Fusion Transcripts

**DOI:** 10.1002/cnr2.70694

**Published:** 2026-10-23

**Authors:** Atbin Latifi, Sina Yousefian

**Affiliations:** ^1^ School of Medicine Arak University of Medical Sciences Arak Iran; ^2^ Student Research Committee Arak University of Medical Sciences Arak Iran

**Keywords:** ALL, CML, drug resistance, neoplasm, neoplasms, Philadelphia chromosome, second primary, tyrosine kinase inhibitors

## Abstract

**Background:**

The sequential emergence of chronic myeloid leukemia (CML) following Philadelphia‐positive acute lymphoblastic leukemia (ALL) is exceedingly rare, and its biological basis remains uncertain.

**Case:**

We report a 15‐year‐old boy initially diagnosed at age 4 with Philadelphia‐positive pre‐B ALL expressing p190 BCR::ABL1. He achieved deep molecular remission following chemotherapy per the COG AALL0232‐based high‐risk B‐ALL protocol plus imatinib. Approximately 5 years after his last documented follow‐up, he re‐presented with splenomegaly, leukocytosis, anemia, and thrombocytosis. Bone marrow findings supported chronic‐phase CML, confirmed by detection of BCR::ABL1 p210 (b2a2 and b3a2) with no detectable p190. Responses to imatinib and dasatinib were suboptimal, and kinase‐domain analysis identified a rare ABL1 c.1159T>A (p.Leu387Met) variant. He subsequently underwent matched‐related allogeneic hematopoietic stem cell transplantation and remains BCR::ABL1‐negative on nilotinib.

**Conclusion:**

This case expands the spectrum of sequential Philadelphia‐positive leukemias by documenting p190‐positive pre‐B ALL followed by p210‐positive chronic‐phase CML, accompanied by detection of the rare L387M variant and suboptimal responses to imatinib and dasatinib. The biological relationship between the two Philadelphia‐positive malignancies remains uncertain, with possible explanations including evolution from a shared Philadelphia‐positive precursor, therapy‐related leukemogenesis, or an independent second malignancy. This case provides unique insight into the biological complexity of sequential Philadelphia‐positive leukemias and adds to the evolving literature regarding the clinical significance of the L387M variant in TKI response. The unsupervised TKI interruption underscores that any consideration of treatment discontinuation should occur only under specialist supervision with close molecular monitoring, while the discordance between the observed response and reported L387M sensitivity patterns warrants further investigation.

## 1 Introduction

Acute lymphoblastic leukemia (ALL) and chronic myeloid leukemia (CML) are biologically distinct entities that can both harbor the Philadelphia chromosome, the defining hallmark of CML and an established driver in a subset of ALL cases. The Philadelphia chromosome arises from a reciprocal translocation between chromosomes 9 and 22, generating the *BCR::ABL1* fusion oncogene [1]. The breakpoint within BCR determines the resulting BCR::ABL1 isoform: p210 is the hallmark of CML but is also detected in a minority of Philadelphia‐positive ALL cases, whereas p190 predominates in Philadelphia‐positive ALL [1]. Tyrosine kinase inhibitors (TKIs), which specifically target BCR::ABL1, are the cornerstone of therapy in Philadelphia‐positive leukemias.

Here, we report an exceptionally rare case of CML developing after Philadelphia‐positive pre‐B ALL, characterized by distinct BCR::ABL1 fusion transcripts at separate presentations, detection of the rare ABL1 L387M kinase‐domain variant, and suboptimal responses to sequential first‐ and second‐generation TKIs.

## 2 Case

In February 2015, a 4‐year‐old boy presented to Amirkabir Hospital in Arak, Iran, with a 4‐week history of fever, malaise, and a palpable left abdominal mass. Examination revealed fever, nontender cervical lymphadenopathy, and splenomegaly extending 5 cm below the costal margin. There was no significant past medical or family history of hematologic or other major diseases. Initial laboratory investigations showed leukocytosis, anemia, and thrombocytopenia (Table 1). Bone marrow aspirate (BMA) contained approximately 90% blasts. Flow cytometry confirmed B‐lineage commitment, with blasts expressing B‐cell antigens while lacking myeloid or T‐lineage markers (Table 2), thereby excluding mixed‐phenotype acute leukemia. Reverse‐transcription PCR detected the BCR::ABL1 p190 transcript, confirming Philadelphia‐positive pre‐B ALL, and cytogenetic analysis demonstrated isolated t(9;22)(q34;q11) without additional abnormalities.

**TABLE 1:** Hematologic findings at initial diagnosis and re‐presentation.

	**I**nitial presentation (pre‐B ALL)	Re‐presentation (CML)
WBC (/μL)	16 000	451 000
Differential (%)	Neutrophils: 10 Lymphoblasts: 80 Monocytes: 7 Eosinophils: 2 Bands: 1	Neutrophils: 29 Lymphocytes: 9 Monocytes: 4 Bands: 12 Eosinophils: 5 Basophils: 8 Myelocytes: 14 Metamyelocytes: 12 Promyelocytes: 4 Blasts: 3
Hemoglobin (g/dL)	6.5	8.4
Platelets (/μL)	6000	1 282 000

*Note:* Comparison of hematologic parameters at initial diagnosis (Philadelphia‐positive pre‐B ALL) and at re‐presentation (CML). At diagnosis, findings included leukocytosis, anemia, and thrombocytopenia, while re‐presentation demonstrated leukocytosis with a full spectrum of myeloid precursors, anemia, and thrombocytosis—*consistent with* the distinct lineage phenotype observed during the later CML presentation.

**TABLE 2:** Flow cytometry findings at initial diagnosis and subsequent CML presentation.

Marker	Philadelphia‐positive pre‐B ALL (diagnosis)	CML presentation
B‐cell	CD10+, CD19+, CD22+, CD79a+, TdT+	CD10 < 1%, CD19 2%, CD20 2%, CD22 NR, CD23 NR, FMC7 NR
T‐cell	CD3—, CD7—	CD2 12%, CD3 11%, CD7 10%, CD1a NR
Myeloid	CD13—, CD33—, MPO—	CD13 65%, CD33 78%, CD117 42%, MPO NR, CD14 NR, CD64 NR
Stem/immature	CD34+, HLA‐DR+	CD34 20%, HLA‐DR NR, TdT NR
Other	CD38+	CD56 9%, CD38 88%, CD45 98%, CD57 NR, CD11c NR

*Note:* Immunophenotypic findings at initial diagnosis (Philadelphia‐positive pre‐B ALL) and at re‐presentation (CML). At diagnosis, blasts expressed B‐cell markers without myeloid or T‐lineage antigens. At re‐presentation, the immunophenotype demonstrated predominant myeloid differentiation with low‐level B‐ and T‐lineage marker expression.

Abbreviation: NR, markers that were not requested in the original laboratory assessment.

The patient received the Children's Oncology Group (COG) AALL0232‐based high‐risk B‐ALL protocol plus imatinib (340 mg/m^2^/day). After 1 month, next generation sequencing (NGS)‐based minimal residual disease (MRD) was < 0.001%. By 6 months, a complete cytogenetic response had been achieved, with BCR::ABL1 PCR decreasing to 0.46%, and at 12 months, BCR::ABL1 had further decreased to 0.08%. Therapy was continued for 3 years, by which time deep molecular remission had been achieved, with undetectable BCR::ABL1 by PCR and NGS‐based MRD < 0.001%. Imatinib monotherapy was then continued for an additional 3 years. At the last documented follow‐up, clinical assessment remained unremarkable and BCR::ABL1 remained undetectable. No hematopoietic stem cell transplant (HSCT) was performed, consistent with standard practice for pediatric Philadelphia‐positive pre‐B ALL in first remission without adverse features [2].

Imatinib was planned for 5–10 years following chemotherapy completion. During imatinib monotherapy, however, follow‐up became irregular due to socioeconomic constraints, with visits occurring annually. At each visit, clinical assessment remained unremarkable and BCR::ABL1 PCR was undetectable. According to the family, the patient discontinued imatinib approximately 3 years into imatinib monotherapy without physician supervision and outside any planned treatment‐free remission strategy. He subsequently ceased attending follow‐up, and no further molecular monitoring was documented until re‐presentation. Because the discontinuation date was based solely on family report, the exact duration of TKI interruption and the timing at which the later Philadelphia‐positive disease emerged could not be reconstructed.

Approximately 5 years after his last visit, the patient re‐presented with malaise and a progressively enlarging abdominal mass over 3 months. Examination revealed massive splenomegaly (15 cm below the costal margin), and laboratory tests showed leukocytosis, anemia, and thrombocytosis (Table 1). Bone marrow examination showed hypercellularity with marked myeloid expansion, and flow cytometry revealed a maturing myeloid pattern with strong expression of myeloid antigens, partial CD56 expression, minimal B‐cell markers, and low‐level T‐lineage antigens (Table 2). PCR detected BCR::ABL1 p210 with both b2a2 and b3a2 transcripts, while p190 was absent, confirming the diagnosis of CML. Karyotyping demonstrated isolated t(9;22)(q34;q11), unchanged from the ALL phase.

Imatinib was restarted at 340 mg/m^2^/day. The patient achieved complete hematologic response at 1 month and complete cytogenetic response at 3 months, with BCR::ABL1 PCR declining to 1.7% (MR1.0). At 6 months, however, the PCR rose to 5.3%—representing an ELN‐defined “warning” response category [3]—prompting a switch to dasatinib. Dasatinib was administered at 70 mg/m^2^/day once daily, according to institutional practice, aiming to balance adequate BCR::ABL1 inhibition with treatment tolerability. On dasatinib, BCR::ABL1 PCR initially decreased to 3.2% (MR1.0) at 3 months but subsequently increased to 8.9% at 6 months, placing the 6‐month response to dasatinib in the ELN warning category.

Bidirectional Sanger sequencing of the BCR::ABL1 kinase domain, performed using cDNA generated from bone marrow RNA, revealed no common resistance‐associated mutations (including T315I, E255K/V, Y253H, V299L, T315A, F359V/C/I, and F317L/V/I/C) but detected a rare heterozygous missense variant, c.1159T>A (p.Leu387Met), within the ABL1 kinase domain. As variants present at frequencies below approximately 15% may not be reliably detected by this method, the presence of low‐level subclones harboring additional resistance‐associated mutations cannot be completely excluded. Given prior reports describing an association between L387M and altered TKI sensitivity, the variant was considered a potentially relevant molecular finding in the context of the observed suboptimal molecular response, although a causal relationship could not be established.

The patient subsequently underwent allogeneic HSCT from a fully HLA‐matched related donor following myeloablative conditioning with intravenous busulfan (0.8 mg/kg/dose every 6 h for 16 doses) and cyclophosphamide (60 mg/kg/day for 2 consecutive days after completion of busulfan). Graft‐versus‐host disease (GVHD) prophylaxis consisted of cyclosporine (5–6 mg/kg/day in two divided doses, adjusted according to therapeutic drug monitoring) and a short‐course methotrexate at 15 mg/m^2^ on Day +1 and 10 mg/m^2^ on Days +3, +6, and + 11. Engraftment was successfully achieved, with recovery of peripheral blood counts, and Day +100 chimerism demonstrated 99% donor cells. No significant acute or chronic GVHD, transplant‐related complications, or other adverse events have been observed during follow‐up. Following engraftment, nilotinib was initiated at 230 mg/m^2^ twice daily because ponatinib was unavailable locally and L387M‐associated resistance to nilotinib had not been established. Treatment was well tolerated, and posttransplant qualitative PCR for BCR::ABL1 remains negative. The longitudinal molecular monitoring and treatment course across both Philadelphia‐positive disease phases are summarized in Figure 1.

**FIGURE 1:**
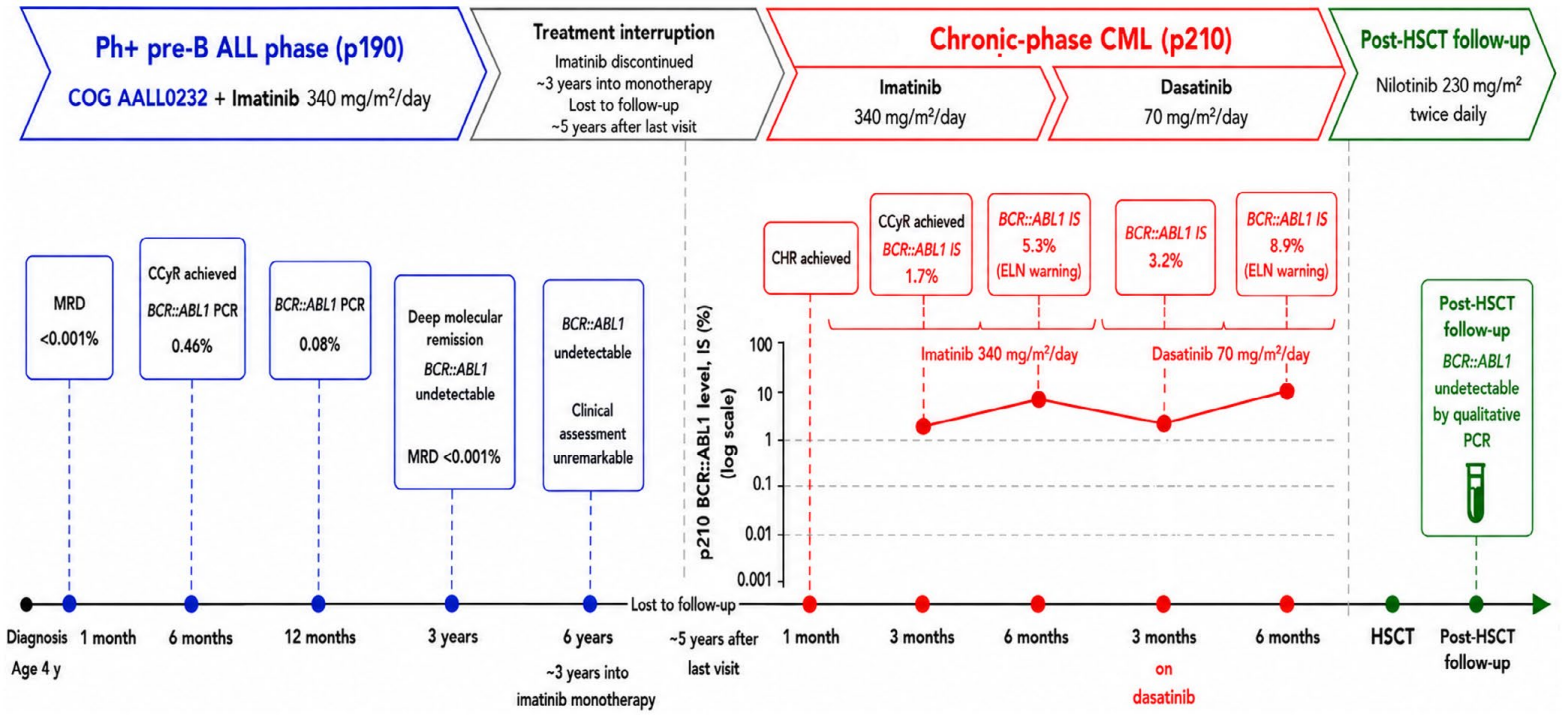
Longitudinal molecular monitoring and treatment course across sequential Philadelphia‐positive leukemias. The blue segment depicts the initial p190‐positive Philadelphia‐positive pre‐B ALL phase treated with the COG AALL0232‐based high‐risk B‐ALL protocol plus imatinib (340 mg/m^2^/day). At 1 month MRD was < 0.001%. At 6 months, complete cytogenetic response was achieved, with BCR::ABL1 PCR decreasing to 0.46%, and at 12 months, BCR::ABL1 further decreased to 0.08%. At completion of 3 years of combined chemotherapy and imatinib, deep molecular remission was achieved, with undetectable BCR::ABL1 by PCR and MRD < 0.001%. Imatinib monotherapy was continued for an additional 3 years; at the last documented follow‐up, clinical assessment remained unremarkable and BCR::ABL1 remained undetectable. The gray segment represents the subsequent unsupervised discontinuation of imatinib and loss to follow‐up. The red segment depicts the later p210‐positive chronic‐phase CML. Complete hematologic response was achieved after 1 month of imatinib. BCR::ABL1 levels on the International Scale were 1.7% at 3 months and 5.3% at 6 months, the latter representing an ELN‐defined warning response. After switching to dasatinib, BCR::ABL1 levels were 3.2% at 3 months and 8.9% at 6 months, representing another ELN warning response. The green segment depicts posttransplant follow‐up, including 99% donor chimerism at Day +100 and persistent qualitative BCR::ABL1 negativity during nilotinib maintenance therapy. ALL, acute lymphoblastic leukemia; CCyR, complete cytogenetic response; CHR, complete hematologic response; CML, chronic myeloid leukemia; ELN, European LeukemiaNet; HSCT, hematopoietic stem cell transplantation; IS, International Scale; MRD, minimal residual disease; NGS, next‐generation sequencing; PCR, polymerase chain reaction.

## 3 Discussion

The sequential emergence of CML following Philadelphia‐positive ALL is exceptionally rare and difficult to distinguish from CML initially presenting in lymphoid blast‐phase, which can mimic de novo Philadelphia‐positive ALL. Several reported cases of apparent “ALL followed by CML” likely represented blast‐phase CML initially misclassified as de novo ALL [4, 5, 6].

Our patient's initial presentation, however, was highly consistent with de novo Philadelphia‐positive pre‐B ALL rather than lymphoid blast‐phase CML, evidenced by the absence of significant leukocytosis, basophilia, eosinophilia, or granulocytic left shift. The immunophenotype was purely lymphoid, and cytogenetics revealed isolated t(9;22) without additional abnormalities typical of CML blast crisis. Importantly, the leukemia expressed only the p190 isoform, strongly associated with de novo Philadelphia‐positive ALL and rarely seen in CML blast‐phase. Finally, the rapid and sustained deep remission following ALL‐directed therapy further supported de novo Philadelphia‐positive ALL, as sustained remissions are uncommon in lymphoid blast‐phase CML.

Well‐documented cases of ALL followed by CML remain exceedingly rare. One child with Philadelphia‐negative pre‐B ALL developed p210‐positive CML after 3 years of remission, a sequence interpreted as compatible with therapy‐related CML (t‐CML) and responded well to imatinib [7]. In another report, an adolescent with pre‐B ALL initially expressing both p190 and p210 BCR::ABL1 transcripts subsequently developed p210‐positive chronic‐phase CML. Molecular analyses demonstrated a shared clonal origin, with both transcripts originating from a single BCR::ABL1 genomic rearrangement through alternative splicing. This report established that transcript and phenotypic divergence may occur within a common Philadelphia‐positive precursor [8]. Unlike these reports, our case was additionally characterized by the rare ABL1 L387M kinase‐domain variant and suboptimal responses to sequential first‐ and second‐generation TKIs, raising a distinct question regarding the clinical significance of L387M in the context of TKI response.

The biological relationship between the two presentations cannot be determined from the discordant fusion transcripts alone, and several possibilities remain. One possibility is evolution from a shared Philadelphia‐positive precursor. This could involve selection and expansion of a Philadelphia‐positive subclone with different biological characteristics, acquisition of additional genomic alterations by the original Philadelphia‐positive precursor, or altered BCR::ABL1 transcript processing, including alternative splicing or differential isoform expression. Therapy‐driven selective pressure may allow genetically or epigenetically distinct subclones to preferentially survive treatment and expand [9], although whether such mechanisms contributed to the present case cannot be determined. In addition, the shared clonal origin demonstrated by Winter et al. supports the biological plausibility of transcript‐level divergence within a common precursor [8].

A second possibility is t‐CML following prior cytotoxic exposure. Although uncommon, t‐CML has been reported after treatment for other malignancies, notably ALL/B‐ALL [10]. Alkylating agents and topoisomerase II inhibitors are most strongly implicated, although the relative risk of subsequent CML remains modest [10, 11]. t‐CML typically develops 4–7 years after treatment and closely resembles de novo CML, most often presenting in chronic phase with isolated t(9;22) and comparable TKI responses [10, 12]. Our patient received both alkylating agents and topoisomerase II inhibitors (cyclophosphamide, daunorubicin, doxorubicin), and the latency interval was consistent with published data, supporting the plausibility of a therapy‐related process.

A third possibility is that the later p210‐positive CML represented an independent Philadelphia‐positive malignancy unrelated to the initial ALL. Thus, the discordant fusion transcripts indicate an unusual sequential disease course but do not prove either a shared or separate clonal origin.

Separate from the uncertainty regarding clonal origin, the L387M variant warrants consideration in relation to the patient's TKI response. L387M is a rare BCR::ABL1 kinase‐domain missense variant (c.1159T>A, NM_005157.6) located within the activation loop. Absent from population databases such as gnomAD and ClinVar, L387M appears in COSMIC/CIViC as a variant of uncertain significance with mixed resistance data. Experimental Ba/F3 models have demonstrated modest imatinib resistance with preserved dasatinib/nilotinib sensitivity [13]. Clinically, L387M has also been identified among kinase‐domain variants detected in patients with imatinib‐resistant CML, although its independent contribution to treatment response remains uncertain [14, 15]. Unexpectedly, our case demonstrated suboptimal responses to both imatinib and dasatinib, representing a clinical response pattern that was not entirely concordant with the previously reported sensitivity profile of L387M. Additional molecular or disease‐related factors may have contributed to this observation. This highlights the need for cautious interpretation of rare kinase‐domain variants, as clinical behavior may not always align with available experimental data.

This case has several important limitations. Archived diagnostic material was unavailable for paired high‐sensitivity genomic analysis across disease phases; consequently, the clonal relationship between the initial ALL and subsequent CML could not be established. Interpretation is further limited by the unsupervised discontinuation of imatinib and subsequent loss of molecular surveillance. As this was not a planned or monitored treatment‐free remission attempt, neither the timing of emergence of the later Philadelphia‐positive disease nor any effect of TKI withdrawal on clonal dynamics can be determined. The temporal association therefore does not establish that discontinuation caused the subsequent CML or that continued therapy would have prevented it, but reinforces that TKI discontinuation should occur only under specialist supervision with close molecular monitoring. Additionally, kinase‐domain analysis was performed using bidirectional Sanger sequencing, which may not reliably detect variants present at frequencies below approximately 15%; therefore, low‐frequency resistance‐associated subclones could have escaped detection, and the variant allele frequency of L387M could not be reliably quantified.

## 4 Conclusion

This case documents an exceptionally rare occurrence of chronic‐phase CML following Philadelphia‐positive pre‐B ALL, characterized by distinct BCR::ABL1 fusion transcripts, detection of the rare ABL1 L387M variant, and suboptimal responses to sequential TKI therapy. The biological relationship between the two Philadelphia‐positive malignancies remains uncertain, with possible explanations including evolution from a shared Philadelphia‐positive precursor, therapy‐related leukemogenesis, or an independent second malignancy. It highlights that a subsequent Philadelphia‐positive disease should undergo complete morphologic, immunophenotypic, cytogenetic, and molecular reassessment rather than automatically being assumed to represent relapse of the original leukemia. The unsupervised interruption of imatinib further emphasizes that any attempt at treatment‐free remission should occur only within a structured program with close molecular surveillance. Finally, the discordance between the observed imatinib and dasatinib responses and previously reported L387M sensitivity profiles suggests that the clinical significance of this variant may be context‐dependent and warrants further functional investigation.

## Author Contributions

**Atbin Latifi:** conceptualization, investigation, writing – original draft, methodology, validation, visualization, writing – review and editing, software, formal analysis, project administration, supervision, data curation, resources. **Sina Yousefian:** conceptualization, resources, data curation, project administration, formal analysis, writing – review and editing, visualization, validation, methodology, writing – original draft, investigation.

## Funding

The authors have nothing to report.

## Ethics Statement

The study was approved by Arak University of Medical Sciences Ethics Committee (date: 2024/12/08, number: IR.ARAKMU.REC.1403.279).

## Consent

Written informed consent was obtained from the patient's parents, and assent was obtained from the patient for publication of this case and any accompanying images.

## Conflicts of Interest

The authors declare no conflicts of interest.

## Data Availability

The data that support the findings of this study are available from the corresponding author upon reasonable request.
